# Role of Reconstructive Microsurgery in Tubal Infertility in Young Women

**DOI:** 10.3390/jcm9051300

**Published:** 2020-05-01

**Authors:** Sorin Barac, Lucian Petru Jiga, Andreea Rata, Ioan Sas, Roxana Ramona Onofrei, Mihai Ionac

**Affiliations:** 1 Victor Babes University of Medicine and Pharmacy, Division of Reconstructive Microsurgery, Clinic of Vascular Surgery, Pius Brânzeu Emergency Clinical County Hospital, Timișoara 300041, România; sorinbarac@gmail.com (S.B.); mihai.ionac@gmail.com (M.I.); 2 Department of Plastic, Reconstructive and Aesthetic Surgery, Hand Surgery, Evangelic University Hospital, Oldenburg 26122, Germany; jigalucian@gmail.com; 3 Victor Babes University of Medicine and Pharmacy, 2nd Clinic of Obstetrics and Gynecology, Timișoara 300041, România; sasioan56@yahoo.com; 4 Victor Babes University of Medicine and Pharmacy, Department of Rehabilitation, Physical Medicine and Rheumatology, Timișoara 300041, România; onofrei.roxana@umft.ro

**Keywords:** infertility, microsurgery, tubal reversal, pregnancy

## Abstract

Aim: Here, we retrospectively analyzed the success rate of reconstructive microsurgery for tubal infertility (RMTI) as a “first-line” approach to achieving tubal reversal and pregnancy after tubal infertility. Patients and Methods: During 9 consecutive years (2005–2014), 96 patients diagnosed with obstructive tubal infertility underwent RMTI (tubal reversal, salpingostomy, and/or tubal implantation) in our centre. The outcomes are presented in terms of tubal reversal rate and pregnancy and correlated with age, level of tubal obstruction, and duration of tubal infertility. Results: The overall tubal reversal rate was 87.56% (84 patients). The 48-month cumulative pregnancy rate was 78.04% (64 patients), of which seven ectopic pregnancies occurred (8.53%). The reversibility rate for women under 35 yo was 90.47%, with a birth rate of 73.01%. The reconstruction at the infundibular segments favored higher ectopic pregnancy rates (four ectopic pregnancies for anastomosis at infundibular level—57.14%, two for ampullary level—28.57%, and one for replantation technique—14.28%), with a significant value for *p* < 0.05. Conclusions: In the context of IVF “industrialization”, reconstructive microsurgery for tubal infertility has become increasingly less favored. However, under available expertise and proper indication, RMTI can be successfully used to restore a woman’s ability to conceive naturally with a high postoperative pregnancy rate overall, especially in women under 35 yo.

## 1. Introduction

Requests for renewing fertility—after consented tubal ligation—arise as a consequence of partner change, improved financial status or, less often, the death of a child. Under these circumstances, assisted reproductive technologies (ART) or tubal microsurgical reconstruction are the two efficient, yet different—in terms of approach, indications, success rates, and complications—tools that can enable pregnancy [1,2]. 

In vitro fertilization (IVF) requires long periods of time, a complex team and serious amounts of both emotional and financial involvement with each embryo transfer [3,4]. In addition to being unable to conceive naturally, the patients undergoing IVF are at high-risk of developing multiple pregnancies across all age groups if they do not choose single embryo transfer [5]. By using reconstructive microsurgery for tubal infertility (RMTI) women are able to conceive naturally more than once if they want.

Women undergoing RMTI are facing other risks involving open surgery, general anaesthesia with hospital stay, and tubal re-occlusion. However, all these are compensated by the prospect of spontaneous pregnancy after natural intercourse, very low risk of multiple pregnancies (<2%), and the possibility to freely decide if the couple will desire another child before considering contraceptive measures [6]. RMTI can yield much higher rates of pregnancy (up to 89%) [1], however, these results are significantly dependent on the patient’s age, tubal obstruction aetiology, ligation method, and type of reversal surgery used.

In this retrospective cohort study, we assessed the efficacy of RMTI, using pregnancy as the main end-point and correlating the success rates with age and level of tubal obstruction. 

## 2. Experimental Section

### 2.1. Patients 

Ninety-six consecutive patients with a mean age of 31.8 years old were referred to our service during 2005–2014 for tubal reversal in the presence of infertility of obstructive aetiology. All patients had their tubes ligated at their own will. Patients’ demographics are detailed in Table 1. Study database is supplementary material to this paper (Table S1). Tubal obstruction was diagnosed either on clinical examination (e.g., previous tubal sterilization) or hysterosalpingography (HSP). Adequate assessment of the ovarian function using detailed transvaginal ultrasound examination and hormone check-up was performed in all patients preoperatively. All male partners performed a spermogram and were within the normal limits as defined by World Health Organization (WHO) [6]. Successful RMTI was defined by evidence of tubal permeability by HSP at one month after surgery, followed by a physiologically obtained pregnancy within 48 months postoperatively. We ruled out other causes of female infertility. Within this period, none of the patients addressed other ART procedures. All patients signed an informed consent for the treatment and for the later use of their clinical files under proper anonymization. The study has the agreement from the Ethics Committee (no. 164/3 July 2019 revised) under the EU GCP Directives, International Conference of Harmonization of Technical Requirements for Registration of Pharmaceuticals for Human Use (ICH), and Declaration of Helsinki.

### 2.2. Surgical Techniques

All surgeries were performed by three microsurgeons (SB, LJ, and MI). Under general anaesthesia, all patients were fitted with a Schultze’s cannula placed transcervically and attached, through specific tubing, to a 50 mL syringe containing diluted Povidone-Iodine (1:4 dilution ratio using normal saline). With the patient in Trendelenburg position, a transperitoneal approach, through a standard minimal (5–7 cm) transverse laparotomy (modified Pfannenstiel with no section of the distal rectus muscles) was used in all patients. 

Detailed inspection of the uterus, adnexa, and ovaries followed. Adherent bands (e.g., because of inflammatory disease or previous surgery) present at this level were carefully divided.

From this point forward, all the surgery continued using an OPMI Vario/S88 Zeiss operative microscope and standard microsurgical techniques.

After completion of RMTI, the abdominal wall was closed using Monomax^®^ 1-0 loop suture (BBraun, Hessen, Germany). Skin was approximated using 4-0 polydioxanone intradermal suture. Routinely, drainage of either the peritoneal cavity and/or subcutaneous space was employed only in selected patients.

#### 2.2.1. Microsurgical Tubal Reversal 

The entire affected scarred tubal segment was resected. The remaining tubal stumps were prepared free of the broad ligaments and haemostasis was achieved by soft bipolar coagulation along the tubal vascular pedicles. We excised an average of 1 cm long from the tube in order to regain healthy tissue to be anastomosed. The permeability of the remaining segments was inspected by injection of diluted povidone-iodine solution transcervically, via the Schultze’s cannula (for the proximal segment) and directly by transtubal cannulation with a 24 G cannula (for the distal segment). 

**Figure 1:**
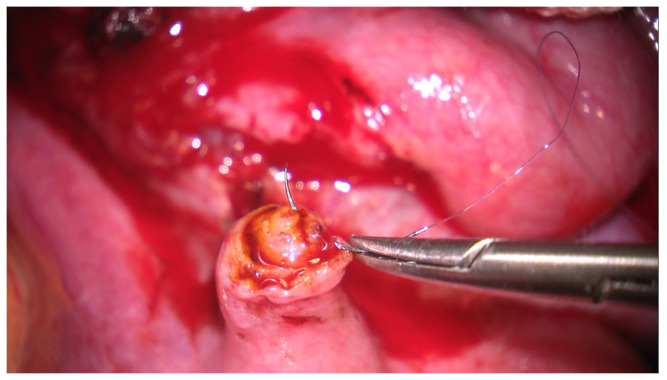
The left proximal tubal extremity can be observed after the sectioning of ligature (in the isthmic region at 2 cm from the uterus) and its preparation from the serosa. One can also notice on the surface of the tubal edge the solution of povidone iodine after testing and the placement of the first wire outside of the mucosal layer. The tube calibre is about 1 mm (zoom × 10).

To approximate the tubal stumps, in all cases, 4–7 separate sutures of 7-0 polydioxanone were placed circumferentially through the muscular layer, avoiding the mucosa (Figure 1). 

**Figure 2:**
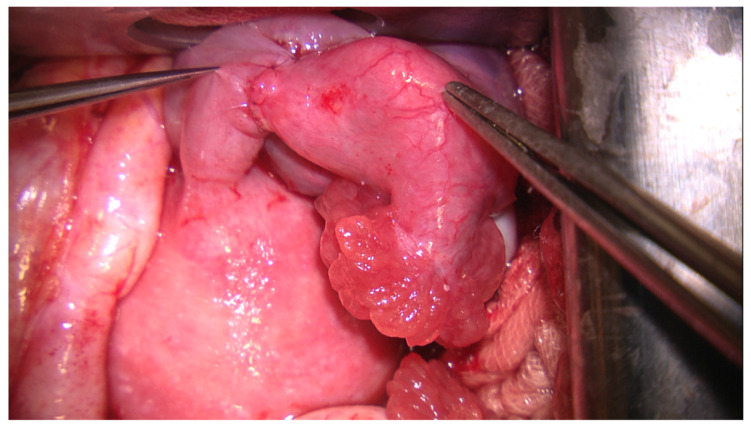
The complete restoration of the tubal anatomy, suture of the peritoneal serosa after the termino-terminal anastomosis (zoom × 10).

The permeability of the tubal lumen was continuously checked during suture. A separate layer of the same suture was used in a continuous manner to approximate the serosa and the divided broad ligament at the level of tubal reconstruction (Figure 2). Finally, tubal permeability was checked again (see Additional Supporting Online Video—Video S1).

#### 2.2.2. Tubo-Cornual Anastomosis

After identifying the scarred area, we resected the uterine tube, then incised the peritoneal serosa and the myometrium (with careful haemostasis with the bipolar cautery). The intramural tube segment was sectioned longitudinally, and the lumen permeability checked. The tubal stump was then incised in a spatulated manner and the suture of the two segments was realized with separate wires, the first two—one at the heal and one at the top—for calibrating the width of the anastomosis; then, the suture was also made with separate wires around the edges in an extramucous manner. The technique combines features from the anastomotic techniques in vascular surgery (termino-terminal spatulated anastomosis in vessels with different calibres) and the techniques described by McComb and Fayez [7,8].

#### 2.2.3. Microsurgical Neosalpingostomy

This technique was employed in all patients presenting with tubal obstruction at the ampulla level with consequent fimbriae atrophy and proximal hydrosalpinx. The fimbriae remnants were carefully excised. A longitudinal 2.5–3 cm incision over the most dilated portion of the proximal segment of the uterine tube was performed. The tip of the neosalpingostomy was fashioned in a cobra-head manner. The tubal lumen was opened over the entire incision length and the mucosal layer was sutured to the tubal wall circumferentially in an everted fashion using continuous 7-0 polydioxanone suture. Tubal permeability was checked again (see Additional Supporting Online Video).

### 2.3. Postoperative Follow-Up

Patients received a short 24-h course of antibiotics and were discharged on postoperative day 3. Check-up of tubal permeability was performed by HSP, 40 days after RMTI, during which sexual intercourse was forbidden. A normal postoperative HSP was interpreted as successful microsurgical reversal and patients were referred back to their gynaecologists for further assistance in obtaining a normal pregnancy.

### 2.4. Statistical Analysis

When appropriate, values were presented as average ± SEM (standard error). Values were compared with MedCalc Statistical Software version 19 (MedCalc Software bvba, Ostend, Belgium). Differences were considered statistically significant at *p* < 0.05. We calculated the Spearman correlation coefficient and 95% confidence intervals (95% CI) for every parameter. The related χ^2^ statistical values were also calculated. Cox’s proportional hazards model was applied to check the association between parameters.

## 3. Results

RMTI consisted of 185 microsurgical tubal reversals: 128 termino-terminal tubal anastomoses at ampullary level (69.18%), five anastomoses at infundibular level (2.7%), and 52 tubal replantations (28.10%). To improve tubal anatomy, additional adhesiolysis between the tubal segments, uterine body and lombo-ovarian ligaments were performed in seven patients. The duration of surgery was 1.2 ± 0.7 h. No major complications were encountered. In two patients, one hematoma and one infection of the surgical access site were declared minor complications and resolved with open drainage and proper antibiotherapy followed by definitive wound closure.

**Figure 3:**
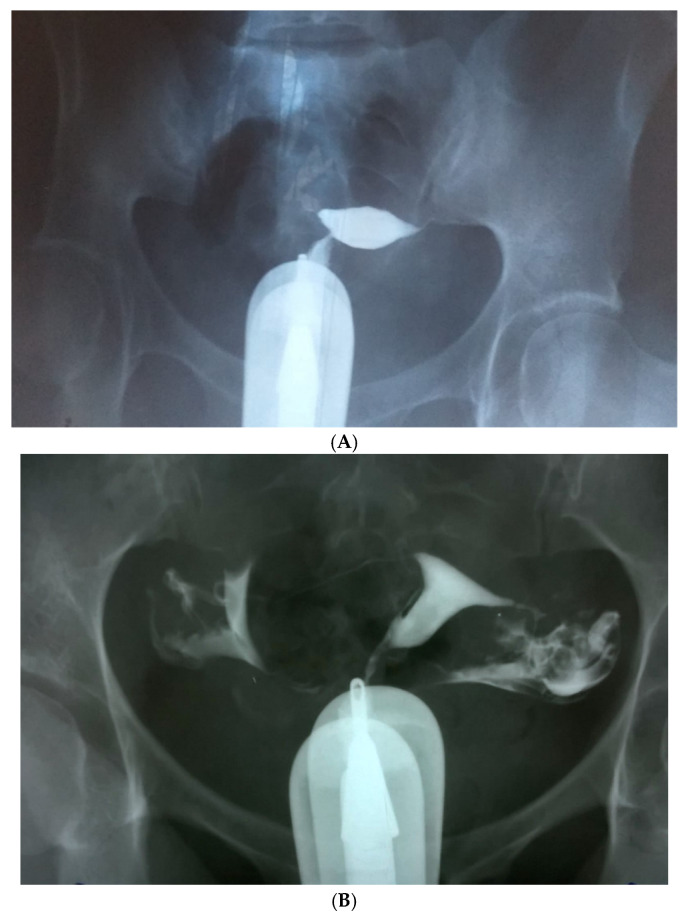
(**A**). Fallopian tube ligated in the cervix-isthmus junction on both sides, after tubal ligation. Our protocol of HSP: 50 mL Iohexol (Omnipaque^®^—GE Healthcare AS Nycoveien Oslo Norway, 240 mg/mL, 0.5 L osmolality Osm/kg H_2_O 37EC, 3.3 mPaxs viscosity, with direct exposure, without delay) were injected with a Schultze device placed transcervical on an automated syringe—Department of Vascular Surgery and Reconstructive Microsurgery collection. (**B**). HSP one month after tubal reconstruction. The passage of the contrast solution through the anastomotic zone until tubal fimbriae can be seen. The image shows uterine tubes completely permeable—Department of Vascular Surgery and Reconstructive Microsurgery collection.

The overall tubal reversal rate, as proven by postoperative HSP was 87.56% (162 out of 185 tubes were reversed as follows: in 79 patients two tubes were reversed and in five patients, only one, respectively) (Figure 3). The 48-month cumulative pregnancy rate was 78.04%, with seven ectopic pregnancies (8.53%). 

With respect to age, the studied parameters (reversibility rate, pregnancy rate and birth rate) were as follows: for the reversibility rate (<35–90.47%, 36–39–83.33%, 40–45–66.67%), pregnancy rate (<35–80.95%, 36–39–60%, 40–45–33.34%), and for the birth rate (<35–73.01%, 36–39–56.67%, 40–45–33.34%).

From Table 2, we can observe that, for the entire sample, we obtained direct and statistically significant correlations between reversibility rate and pregnancy rate (0.549, *p* < 0.0001), between reversibility rate and birth rate (0.468, *p* < 0.0001) and between pregnancy rate and birth rate (0.762, *p* < 0.0001). We also find indirect and statistically significant correlations between age and pregnancy rate (−0.234, *p* < 0.05), reversibility rate and ligation level (−0.399, *p* < 0.05), pregnancy rate and ligation level (−0.577, *p* < 0.0001) and birth rate and ligation level (−0.615, *p* < 0.0001).

Age proved to be a major determinant both of reversibility rate and of pregnancy. In the group of 40–45 yo, only one patient gave birth (Table 3, Table 4 and Table 5).

For the group under 35 yo, we found direct and statistically significant correlations for reversibility rate and pregnancy rate (0.612 with *p* < 0.0001), reversibility rate and birth rate (0.502, *p* < 0.000), and pregnancy rate and birth rate (0.659, *p* < 0.0001). We also found indirect and statistically significant correlations between reversibility rate and ligation level (−0.433, *p* < 0.05), pregnancy rate and ligation level (−0.518, *p* < 0.0001), and between birth rate and ligation level (−0.602, *p* < 0.0001).

For the group aged 35–39, we found statistically significant correlations between pregnancy rate and birth rate (direct correlation −0.916, *p* < 0.0001) and between ligation level and pregnancy rate (indirect correlation −0.674, *p* < 0.05), and also birth rate (indirect correlation, −0.607, *p* < 0.05).

In the group of patients aged 40+ we found no significant correlation between any parameters. 

Moreover, the level of reconstruction influenced the reversal rate. The reconstruction at the fimbrial segments favoured higher ectopic pregnancy rates (4 ectopic pregnancies for anastomoses at infundibular level—57.14%, 2 for ampullary level—28.57% and 1 for replantation technique—14.28%), with a significant value for *p* < 0.01.

The results of the logistic regression analysis (Table 6) show that the model was statistically significant (χ^2^ = 67.820, df = 4, *n* = 96, *p* < 0.001). The model classified correctly 89.58% of the cases. The ligation level contributed significantly, with an inverse correlation to the occurance of birth.

## 4. Discussion

The important aspects that affect the outcome in the case of sterilization reversal (in the absence of a male infertility factor) are the age of the woman, level of tubal reconstruction and a good microsurgical technique. Our study did not take under consideration the male factor because more than 90% of the women involved had a child with the same partner. This aspect could be a limitation of our study.

In our study, the rate of tubal reversal was 87.56%. Yadav et al. reported a rate of success between 50% and 80% when they performed RMTI [3].

All our patients had their tubes ligated as a contraceptive method, after their last birth in an elective manner. Most of them later regretted that decision and wanted to restore their fertility, usually due to changes in socio-economic status or in case of changing the partner and wishing to have a child with this one [4,5]. 

In our study, all male partners had a spermogram within normal values; we did not analyze thoroughly the male factor and this can be considered a limitation of our study.

The tubes were ligated through Pomeroy technique (ligation and section) or only ligated [9,10].

An important fact that needs to be addressed is the awareness of the female population regarding less invasive and radical contraceptive methods.

Pregnancy rate in this study was 78.04%, consistently with the ones found in literature: Hwa Sook Moon—84.7% (from 961 cases, 2012) [11] and Boeckxstaens—59.5% (2007) [12].

The rate of getting pregnant decreases with age: only one pregnancy in women after 40 y.o.; findings are consistent with those of other studies (Kim et al. 1997, Dubuisson and Chapron 1998, Hanafi 2003) [13,14,15]. A large number of studies on tubal reversal also confirmed significantly lower pregnancy rates in older patients [16,17]. Although it is known that fertility rate decreases with age, Trimbos-Kemper, in 1990, evaluated the outcome for RMTI in women aged 40+ and had a 44% birth rate [18].

Another important issue is the level of tubal reconstruction. When the anastomosis is performed closer to the fimbriae, there is a higher risk of ectopic pregnancy. The majority of studies reported a rate of ectopic pregnancies around 20% [19,20]. The findings presented in this study are similar to the ones quoted.

We considered that the path to a higher rate of pregnancy is the microsurgical anastomosis for the Fallopian tubes. RMTI requires a lot of training in order to perform it perfectly with three key points in having a higher rate of success of the tubal recanalization: first—an accurate suture method in order to keep the patency of the tube, second—a perfect alignment of the tubal segment, and third—a proper management of the diameter discrepancy between the two segments that needs to be anastomosed [21].

A topic of discussion is the laparoscopic method; it is a widespread method, but the outcomes are inferior to those of the microsurgical technique, mostly because of the anastomosis procedure. Through this method, the suture is less accurate and conducts to less precision in the anastomosis of the tube because of the wire tension on the stich, the damage of the tubal mucosa, the dissection control, and the bi-dimensional image [22].

The literature data regarding the differences between the robotic technique and the microsurgical one were also analysed. The first technique needs longer surgical and anaesthetic times and the costs are definitely higher. The only positive aspect refers to a shorter period of hospitalization [23].

The second technique requires a longer surgical training period and can be useful in the case of special situations like tubal replantation. The replantation at the cornual level is the most difficult technique for two reasons: the small calibre of the lumen, and the very tiresome anastomosis because of the thick layer of myometrium that obstructs the view of the proximal stump (MRTI is the most suitable due to its higher precision).

Messinger et al. stated, in 2015, that tubal anastomosis was more effective in terms of costs than the IVF procedures, especially in women under 41 [24]. These conclusions come to strengthen those of Boeckxstaens et al. from 2007 who said that IVF is more suitable for women aged 37+ [12].

## 5. Conclusions

After a close analysis of the data and other similar studies, with a 78.04% pregnancy rate, we consider the RMTI as the first line method for sterility reversal in women under 35, having the advantage of an accurate method that requires minimal resources and with low morbidity rates. The alternatives of either tubal reversal or IVF must be decided by the patient (after carefully analysing every aspect of every method) together with her gynaecologist, which is also her primary physician not only until the pregnancy is obtained, but also until successful birth. 

It would be interesting to see randomized trials in order to assess every type of tubal reversal—open microsurgery, laparoscopic surgery and robotic surgery. Of course, more studies are needed to compare all methods in terms of their success. 

## Figures and Tables

**Table 1:** Patients’ demographics.

Parameter		Value (No of Patients)	Percentage (%)
Age (yo)	<35	63	65.62
	35–39	30	31.25
	40–45	3	3.12
Comorbidities	Pelvic inflammatory disease	6	6.25
	Contralateral adnexectomy *	7	7.29
	Uterine leiomyoma	3	5.2
	Uterine malformation (Bicornuate uterus)	1	1.04
Ligation type	Pomeroy technique (ligation and cutting)	84	87.5
	Ligation only	12	12.5
Ligation level	Ampularry	62	64.58
	Infundibular	18	18.75
	Cornual	16	16.67

* Contralateral adnexectomy for ectopic pregnancy and the tubal ligation were performed in the same surgery.

**Table 2:** Correlations between age group and reversibility rate, pregnancy rate, birth rate and ligation level (for the entire sample = 96 patients).

		Ligation Duration	Age	Reversibility Rate	Pregnancy Rate	Birth Rate
Age	Correlation coefficient Significance Level P	0.115 0.2662				
Reversibility rate	Correlation coefficient Significance Level P	0.064 0.5340	−0.186 0.0700			
Pregnancy rate	Correlation coefficient Significance Level P	−0.60 0.5609	−0.234 0.0217	0.549 <0.0001		
Birth rate	Correlation coefficient Significance Level P	−0.009 0.9324	−0.197 0.0538	0.468 <0.0001	0.762 <0.0001	
Ligation level	Correlation coefficient Significance Level P	−0.002 0.9863	−0.020 0.8455	−0.399 0.0001	−0.577 <0.0001	−0.615 <0.0001

**Table 3:** Correlations between age group and reversibility rate, pregnancy rate, birth rate and ligation level for the patients aged under 35.

		Ligation Duration	Age	Reversibility Rate	Pregnancy Rate	Birth Rate
Age	Correlation coefficient Significance Level P	0.136 0.2599				
Reversibility rate	Correlation coefficient Significance Level P	0.101 0.050	−0.056 0.6459			
Pregnancy rate	Correlation coefficient Significance Level P	−0.089 0.4621	−0.044 0.7149	0.612 <0.0001		
Birth rate	Correlation coefficient Significance Level P	−0.005 0.9687	−0.030 0.8080	0.502 <0.0001	0.659 <0.0001	
Ligation level	Correlation coefficient Significance Level P	−0.017 0.8864	−0.122 0.3147	−0.433 0.0002	−0.518 <0.0001	−0.602 <0.0001

**Table 4:** Correlations between age group and reversibility rate, pregnancy rate, birth rate and ligation level for the patients aged 35–39.

		Ligation Duration	Age	Reversibility Rate	Pregnancy Rate	Birth Rate
Age	Correlation coefficient Significance Level P	−0.186 0.3944				
Reversibility rate	Correlation coefficient Significance Level P	−0.127 0.5628	−0.082 0.7098			
Pregnancy rate	Correlation coefficient Significance Level P	0.106 0.6306	−0.055 0.8046	0.388 0.0671		
Birth rate	Correlation coefficient Significance Level P	0.125 0.5704	−0.176 0.4215	0.339 0.1130	0.916 <0.0001	
Ligation level	Correlation coefficient Significance Level P	0.103 0.6412	−0.213 0.3295	−0.283 0.1914	−0.674 0.0004	−0.607 0.0021

**Table 5:** Correlations between age group and reversibility rate, pregnancy rate, birth rate and ligation level for the patients aged 40+.

		Ligation Duration	Age	Reversibility Rate	Pregnancy Rate	Birth Rate
Age	Correlation coefficient Significance Level P	0.500 0.6667				
Reversibility rate	Correlation coefficient Significance Level P	0.866 0.3333	0.000 1.0000			
Pregnancy rate	Correlation coefficient Significance Level P	0.000 1.0000	−0.866 0.3333	0.500 0.6667		
Birth rate	Correlation coefficient Significance Level P	0.000 1.0000	−0.866 0.3333	0.500 0.6667	1.000	
Ligation level	Correlation coefficient Significance Level P	−0.500 0.6667	0.500 0.6667	−0.866 0.3333	−0.866 0.3333	−0.866 0.3333

**Table 6:** Logistic regression with occurrence of birth as a dependent variable.

Independent Variables	b	Standard Error	Wald	*p*	OR	CI
Reversibility rate	1.371	1.757	0.608	0.435	3.938	0.125 to 123.419
Pregnancy rate	3695	0.921	16.068	0.0001	40.246	6.608 to 245.124
Ligation level (fimbriae)	1.875	0.808	5387	0.02	0.153	0.031 to 0.746
Ligation level (cornual)	−2.454	1.107	4.911	0.026	0.085	0.009 to 0.753
Constant	−2.312	1.749	1.747	0.186		
Model χ^2^ = 67.82, *p* < 0.001 *n* = 96						

## Supplementary Materials

The following are available online at https://www.mdpi.com/2077-0383/9/5/1300/s1, Video S1: Permeability test after microsurgical tubal reconstruction, Table S1: Study database.
